# Oxidative stress in prostate hyperplasia and carcinogenesis

**DOI:** 10.1186/s13046-016-0418-8

**Published:** 2016-09-08

**Authors:** Udensi K. Udensi, Paul B. Tchounwou

**Affiliations:** NIH/NIMHD RCMI Center for Environmental Health, College of Science, Engineering and Technology, Jackson State University, Jackson, MS 39217 USA

**Keywords:** Prostate cancer, Oxidative stress, Antioxidants, Cancer treatment

## Abstract

Prostatic hyperplasia (PH) is a common urologic disease that affects mostly elderly men. PH can be classified as benign prostatic hyperplasia (BPH), or prostate cancer (PCa) based on its severity. Oxidative stress (OS) is known to influence the activities of inflammatory mediators and other cellular processes involved in the initiation, promotion and progression of human neoplasms including prostate cancer. Scientific evidence also suggests that micronutrient supplementation may restore the antioxidant status and hence improve the clinical outcomes for patients with BPH and PCa. This review highlights the recent studies on prostate hyperplasia and carcinogenesis, and examines the role of OS on the molecular pathology of prostate cancer progression and treatment.

## Background

Prostate cancer is the most common non-skin cancer affecting men and about 3 million men in the United States (U.S.) are currently living with the disease [1]. It is the second leading cause of cancer death after lung cancer among men in the U.S. [2]. Developing countries share a higher burden of PCa with higher incidence and death rates. It has been reported that PCa is a leading cause of cancer-related deaths among men in Nigeria [3].

PCa is classified as an adenocarcinoma and it is further classified based on its cell of origin. About 95 % of prostate cancers develop in the acini of prostatic ducts. The remaining 5 % are rare histopathologic types which include; small cell carcinoma, mucinous carcinoma, endometrioid cancer (prostatic ductal carcinoma), transitional cell cancer, squamous cell carcinoma, basal cell carcinoma, adenoid cystic carcinoma (basaloid), signet-ring cell carcinoma and neuroendocrine cancer [4]. Risk factors for PCa include; increasing age, family history, genetics, race (African-Americans are mostly affected), dietary factors. Some food nutrients have a level of protection against prostate cancer reduced fat intake, soy protein, lycopene, vitamin E, selenium [5]. Some plants such as *Vernonia amygdalina* have shown to have components which may complement the therapeutic effects of some established PCa drugs such as Paclitaxel [6].

Oxidative stress (OS) is considered to be one of the mechanisms that trigger the chain of reactions involved in the development and progression of prostatic hyperplasia (PH). OS is a condition in the cellular environment which occurs when there is an imbalance between the production of reactive oxygen species (ROS) and the ability of biological systems to repair oxidative damage or neutralize the effects of reactive intermediates including peroxides and free radicals. Production of high levels of ROS causes a significant decrease in antioxidant defense mechanisms leading to protein, lipid and DNA damage and subsequent disruption of cellular functions and cell death but at lower levels induce subtle changes in intracellular signaling pathways [7, 8]. The oxidative damage can be exacerbated by a decreased efficiency of antioxidant defense mechanisms [9]. Like many different cancer types, OS has been linked with benign prostatic hyperplasia (BPH) and prostate cancer (PCa) development, progression and the response to therapy [10–14]. OS and PCa are both associated with increasing age because PCa is more prevalent in older men. Hence, it has been reported that age increases the prooxidant-antioxidant balance toward a more oxidative state in many tissues [15].

**Fig. 1:**
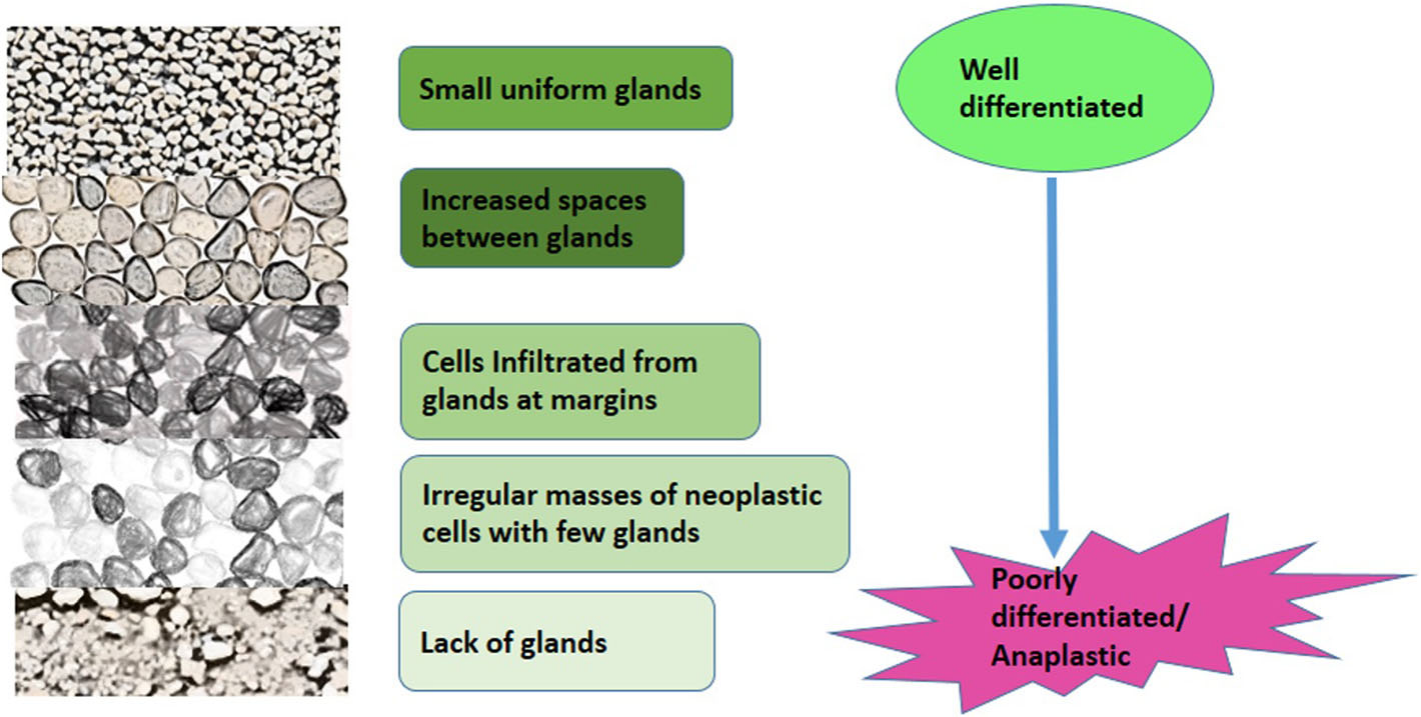
Gleason’s Pattern of Prostate Carcinogenesis: Gleason’s score is the standard used to stage prostate cancer. It helps to determine the treatment strategy to be employed

**Fig. 2:**
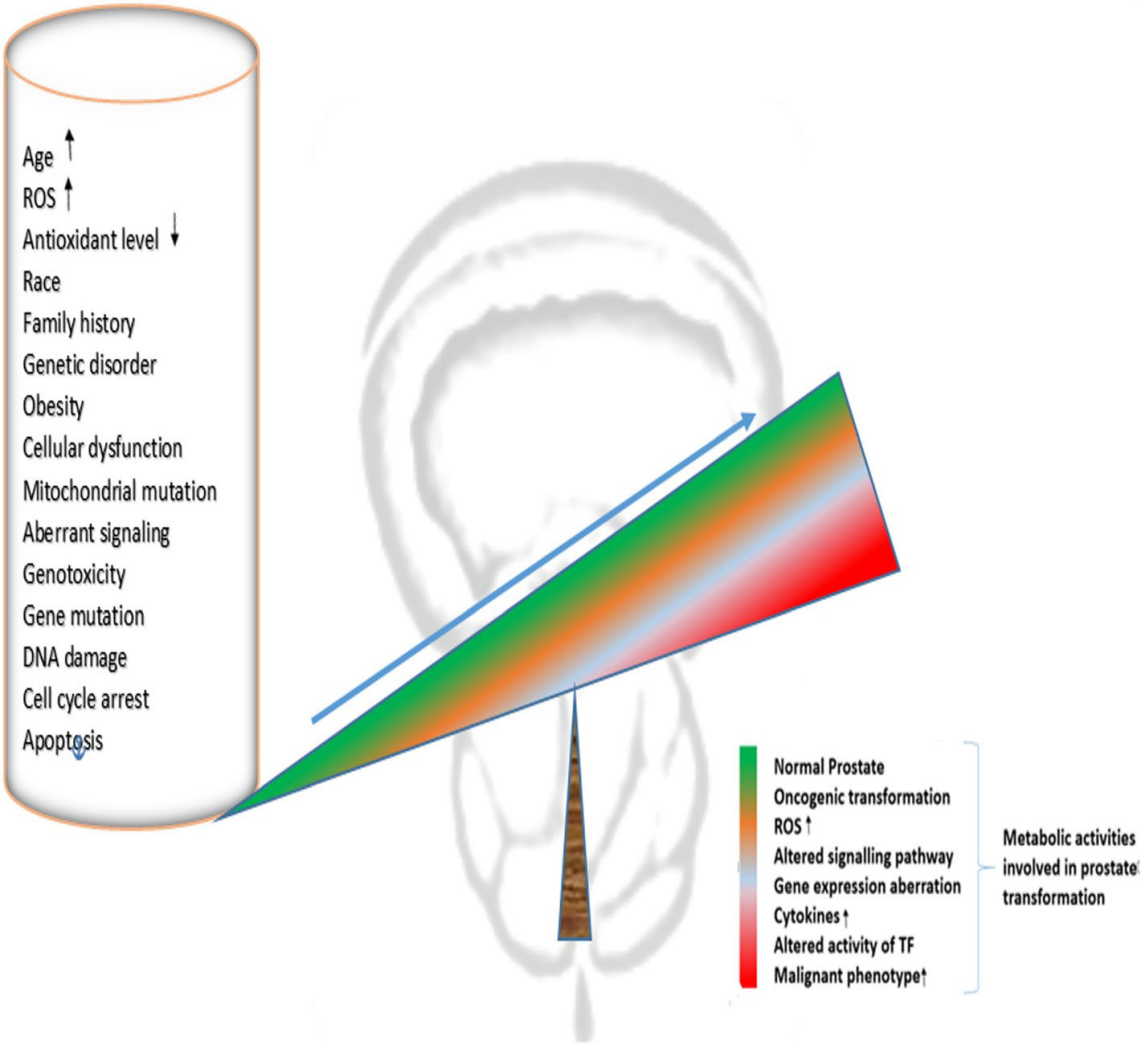
Prostate Cancer and Predisposing Factors: This illustrates the relationship between oxidative stress, antioxidant agents and other predisposing factors such as age, sex, race, and family history in prostate cancer

Several mechanisms for prostate hyperplasia development have been suggested and these include; oxidative stress (OS) [10–14], inflammatory mediators [3, 16–20], hormones (especially androgens whose increase in physiologic level can cause increase in oxidative stress and alterations in intracellular glutathione levels and the activity of other detoxification enzymes required for the maintenance of the cellular prooxidant-antioxidant balance such as gamma-glutamyl transpeptidase) [15], enzymatic factors, dietary factors [21–23], inflammatory genes [17, 24] and Gleason score grading system (Fig. 1) which is used to evaluate the prognosis of PCa [12]. Reactive nitrogen species (RNS) and ROS are byproducts of normal cellular metabolism which impact on cell signaling. Increase in the levels of ROS and RNS induces oxidative stress, causing the cells to activate a variety of mechanisms that allow them to cope with these changes [25]. It is known that OS contributes to the initiation and progression of PCa by regulating molecules such as DNA, transcription factors, and cell cycle regulators [12]. Other studies have shown that antioxidants and other molecules that protect cells against OS play a role in the prevention of PCa. The potential chemoprotective role of ROS regulators in the fight against PCa has been reported [26]. Chronic increases in ROS over time are known to induce somatic mutations and neoplastic transformation [27]. As shown in Fig. 2, several predisposing factors have been postulated to contribute to PCa initiation, promotion and progression. Age, race and family history play predominant roles however environmental factors such as chronic prostatitis, diet, medication and exposure radiation are associated with PCa. Cellular dysfunction including aberrant signaling, genotoxicity, gene mutation, DNA damage, cell cycle arrest, apoptosis and mitochondrial mutation also affect the PCa carcinogenesis and metastasis.

This review highlights recent studies on the role of OS in prostatic hyperplasia initiation, promotion and progression. It also discusses its potential for treatment and offers more insight into the relationships between oxidative stress, redox homeostasis (antioxidants balancing the negative effect of free radicals), growth activation, and induction of molecular pathways in prostate tumorigenesis.

### Prostate cancer and enzymatic antioxidants

PCa is characterized by an increase or decrease in blood levels of certain enzymes and antioxidants. One of such agents is malondialdehyde (MDA) which is a product of the peroxidation of polyunsaturated fatty acids and some esters and it is commonly used as an indicator of lipid peroxidation. Its determination is based on a non-invasive method and its level in blood is measured to determine oxidative stress status of the body. Increase of MDA level is associated with oxidative stress [10, 21, 28–31]. Oxidative stress could also be estimated through measuring the levels of erythrocyte MDA, erythrocyte activities of superoxide dismutase (CuZn-SOD), glutathione peroxidase (GPX), catalase (CAT), plasma nitrite/nitrate (NO(2)(−)/NO(3)(−)), cGMP and 8-hydroxy-2'-deoxyguanosine (8-OHdG) in plasma of prostate cancer patients. PCa is correlated with an imbalance in the oxidative stress/antioxidant status and an alteration of nitrosative status [31, 32].

The application of MDA in PCa diagnosis is growing and its measurement is now done in combination with prostate-specific antigen (PSA) which is a sensitive and generally accepted marker for prostatic hypertrophy and cancer. Increase in PSA is correlated with the severity of PCa but PSA is always performed with another marker such as MDA [21, 28, 33]. Manganese superoxide dismutase (MnSOD) is under consideration as potential clinical marker to predict the progression of PCa [34]. Superoxide dismutase-3 (SOD3) is known to protect cell surface from oxidative stress. It has been reported that the expression of SOD3 is reduced in PCa tissue. Also, an inhibition of cell proliferation, migration, and invasion has been associated with SOD3 overexpression in PC-3 cell line [22]. Generally, PCa is accompanied with a decrease in serum level of anti-oxidants such as GPX, GSH-Px, SOD [14, 35], and an increase in concentrations of thiobarbituric acid reactive substances (TBARS) [36] and lipid peroxidation byproducts [11]. Thioredoxin 1 (Trx 1), is another enzyme which acts as a subcellular indicator of redox status in PCa and has demonstrated that both endogenous and exogenous antioxidants are involved in determining how PCa progresses. It can be correlated with the Gleason score. The level of Trx1 can be used to predict the stages of PCa, the difference between malignant and benign tissue [37]. Nitric oxide generated by inducible nitric oxide synthase (iNOS) may be involved in prostate tumorigenesis, however the exact mechanism has not been elucidated, and warrants further studies [38].

### Prostate cancer and non-enzymatic antioxidants

The idea that OS is involved in prostate tumorigenesis has been supported by the observed decrease in levels of non-enzymatic antioxidants, such as vitamins C and E, in the plasma and erythrocytes of PCa patients compared to normal subjects [39, 40]. As an antioxidant, vitamin E scavenges lipid radicals and terminates oxidative chain reactions by interacting with the lipid peroxyl radical. This prevents further generation a new radical. However, there are contrary opinions about the role of vitamin C and vitamin E in PCa carcinogenesis. The effect of vitamin C is still murky, in-vitro studies with cell line models show that vitamin C can fight against PCa [41] but in-vivo studies could not demonstrate vividly the same result. However, different forms of vitamin E are said to have different effects; alpha-tocopherol which scavenges singlet oxygen potentially increases the risk while gamma-tocopherol potentially decreases risk of developing PCa [41, 42]. PCa patients also have lower levels of zinc (Zn) [11]. The reduction in antioxidants in PCa patients suggests that micronutrient supplementation could be helpful in the prevention and management of the disease [14, 35, 43, 44]. Supplementation should be approached with caution as some micronutrients such as vitamin D may have a detrimental effect and increase the risk of PCa [45]. It has been reported that metastatic PCa patients have a higher Gleason score (*p* < 0.01) and more hormonal treatment, but lower concentrations of PSA (*p* < 0.05), alpha-tocopherol (*p* < or = 0.05), retinol (*p* < 0.01), lutein (*p* < 0.05) and lycopene (*p* < 0.01), compared with patients having localized disease. Lower concentrations of carotenoids, in particular, lycopene reflect disease progression rather than the systemic inflammatory response in patients with PCa [21].

### Oxidative stress, prostate cancer and diet

Diet plays a pivotal role in general body wellbeing. It not only boosts the immune system but also provides antioxidants that help the system to neutralize the negative effects of oxidative stress. Oxidative stress induced by chronic inflammation could be a cause, and dietary intake of antioxidants such as selenium may reduce the risk of developing prostate hyperplasia by reducing the deleterious effects of oxidative stress [46]. Selenium (Se) has been shown to prevent the development of PCa and it is highly accumulated around the prostate gland. Selenoproteins inhibit the transformation of normal prostate epithelium into neoplasm. A reduction in blood level of selenoproteins has been correlated with the risk of PCa [47]. Intake of some food rich in antioxidants can boost body’s protection against disease. Pomegranate, a plant rich in antioxidants, has shown some anti-PCa promises as it slows prostate cancer xenograft growth and prolongs prostate-specific antigen (PSA) doubling times [48]. Extracts from *Vernonia amygdalina* has shown promises a supplementary drug to taxol-resistant prostate adenocarcinoma cells [6].

**Fig. 3:**
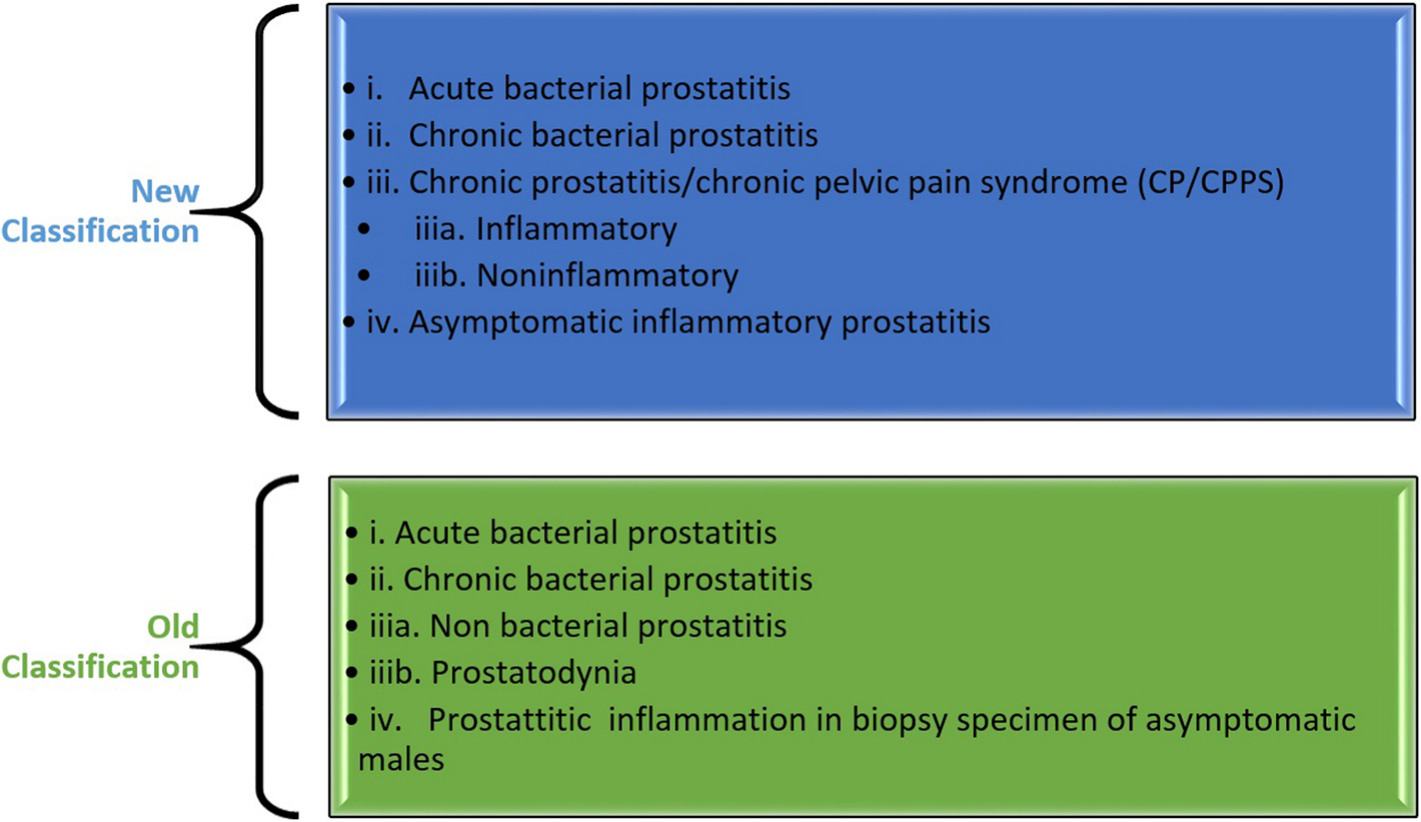
The NIH Consensus Classification of Prostatitis Syndromes [54, 56, 57, 150]

Oxidative stress and body composition contribute to the progression of PCa. High fat diet (HFD) has been identified as a risk factor for PCa because HFD induces oxidative stress and inflammation in the prostate gland. This stress triggers a cascade of activities within the gland culminating to hyperplasia. HFD induces significant increases in the levels of pro-inflammatory cytokines and gene products. It is also speculated that HFD can activate signaling pathways. For example, the signal transducer and activator of transcription (STAT)-3 and nuclear factor-kappa B (NF-kB) which are transcription factors required for regulating genes involved in proliferation, survival, angiogenesis, invasion and inflammation [17]. Measuring body composition can be used to predict risk to developing PCa. This phenomenon has been by using a combination of the measurement of glutathione, fat mass (FM) and waist circumference (WC) to predict the risk of PCa [13]. A study has suggested that dietary fat could encourage increase in proliferation of prostate intraepithelial neoplasia (PIN) and suppression of glutathione peroxidase 3 (GPx3) expression [22]. PIN stage is crucial in prostate carcinogenesis as shown in Fig. 3. Cells have gone through neoplastic changes and have become carcinogenic and ready to invade other cells. High-grade prostatic intraepithelial neoplasia has a high predictive value and is considered as a reliable indicator of pre-invasive stage of adenocarcinoma [49]. An antioxidant, 2-hydroxy-4-methoxy benzoic acid (HMBA) has protective effects in rats against testosterone induced BPH and this may have a similar effect on PCa [29].

### Symptoms and diagnosis of PCa

**Fig. 4:**
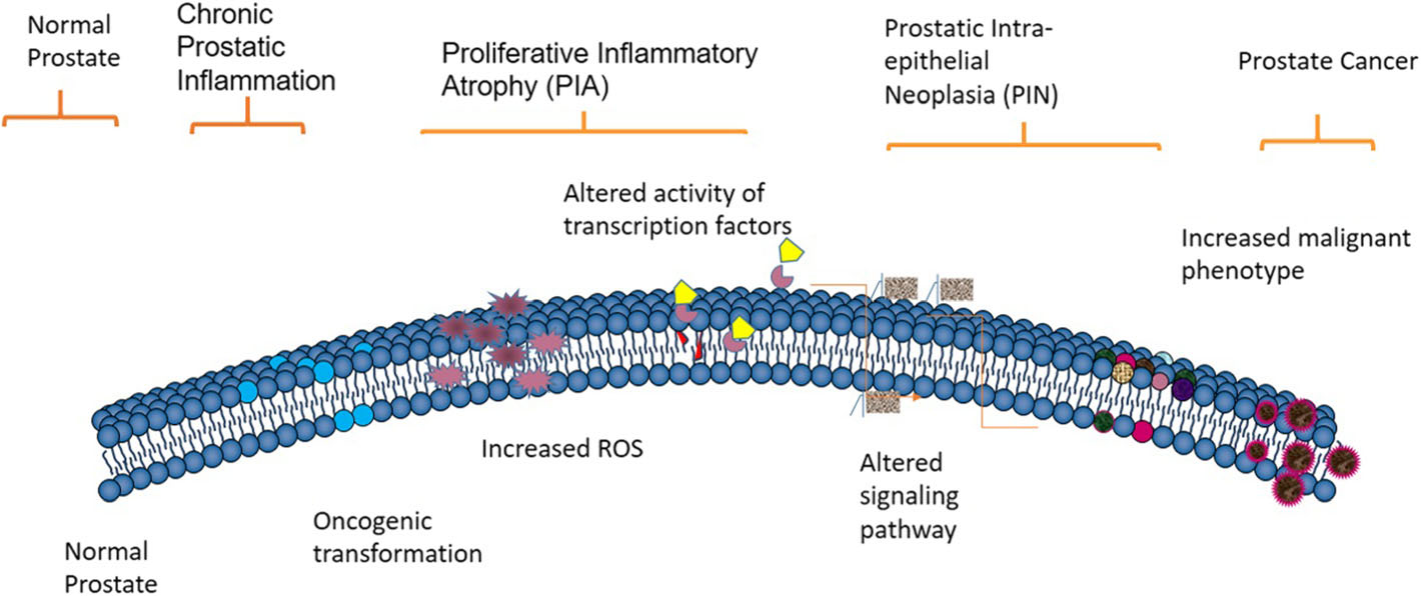
Prostate Carcinogenesis Model: This illustrates what happens at the cellular level as prostate hyperplasia progresses from asymptomatic to metastatic stage

PCa presents different clinical signs and symptoms which range from asymptomatic, inactive, slow-growing tumors to aggressive, fast-growing tumors with lethal progression. Symptoms of PCa may include; problems passing urine, such as pain, difficulty starting or stopping the stream, or dribbling, low back pain and pain with ejaculation. The rate at which cancer grows and the difference in its appearance from surrounding tissue helps determine the stage [50]. Like most epithelial cancers the keys to survival and treatment are early diagnosis and identification of PCa type. Androgens and androgen receptor (AR) are required by both normal prostate and prostate cancer cells for growth and survival [51]. Androgen receptor mutations are observed in late stage prostate cancer. Androgen ablation and antiandrogen therapy cause the cancer to regress. Androgen-independent prostate cancer which does not respond to anti-androgen therapy has been observed in some patients especially in patients whose cancer was not cured by surgery. Overexpression of Caveolin-1 occurs in about a quarter of human prostate cancers and is thought to induce androgen sensitivity in androgen-insensitive prostate cancer cells [52]. However, a recent study has suggested that Metformin, commonly used for type 2 diabetes, may have promising therapeutic effects on both androgen-dependent and androgen-independent PCa [40]. Most facilities use diagnostic test kits that measure the level of prostate-specific antigen (PSA) in serum of patients to detect early stages of PCa. If the PSA level is high, the patient is subjected to more invasive biopsy to ascertain the histopathological grading. A Gleason scoring is used to classify the extent of differentiation of tumors as well as staging (determination of the status of the primary tumors, with or without lymph node involvement) [53]. The categories of prostatitis are shown in Fig. 4.

Prostatitis are classified in to four categories by Meares and Stamey [54], but this was later replaced by the U.S. National Institutes of Health (NIH) classification scheme: Category I-acute bacterial prostatitis; Category II-chronic bacterial prostatitis; Category III-chronic abacterial prostatitis/chronic pelvic pain syndrome [CPPS]) [55]. Category III was further subdivided into IIIa-inflammatory CPPS, and IIIb-non-inflammatory CPPS. Category IV encompasses asymptomatic inflammatory prostatitis. Prostate specimens often reveal evidence of category IV prostatitis after a biopsy [56, 57]. Differential diagnosis of PCa involves differentiating symptoms of PCA; those caused by acute cystitis, benign prostatic hyperplasia (BPH), urinary tract stones, bladder cancer, and rectal tumor and other factors that cause strictures. Other factors that cause urinary symptoms similar to PCa include urinary tract infections (UTI), diuretics, spinal injury, autonomic neuropathy, prostatic abscess, enterovesical fistula, and foreign body within the urinary tract. Most of these conditions like PCa cause urinary obstruction, urine retention, lack of urine, urinary dribbling, urinary urgency, urination pain, and weak urination [58].

The diagnostic methods for PCa include assays based on the evaluation of oxidative stress. The assays are further categorized into: (i) assays that measure the concentrations of oxidation products of lipids, proteins and DNA, as well as the concentrations of antioxidants, (ii) assays that determine the oxidative and reductive capacity of biological fluids, and (iii) assays that measure the ex vivo susceptibility of lipids to oxidation when exposed to a source of free radicals [59]. Some of the oxidative biomarkers that are frequently measured are; lipid peroxidation, total antioxidant capacity and total thiol molecules [60]. Table 1 presents a list of laboratory tests that are used to diagnose and monitor the progress of PCa.

**Table 1:** Laboratory Tests Used to Diagnose and Monitor PCa Biomarkers

Test	Specimen	Factors measured	Method	Reference
*Oxidative stress*	Blood	Activity changes of superoxide-dismutase (SOD), catalase (CAT), ceruloplasmin (Cp), tripeptide glutathione (GSH), glutathione-peroxidase (GSH-Px), and glutathione-reductase (GR)	Spectrophotometry	[11, 12, 32]
*Oxidative stress*	prostate tissue	Thioredoxin 1 (Trx 1)	Spectrophotometry	[37]
*Oxidative stress*	Tissue	Inducible NOS (iNOS or NOS-2)	Immunohistochemistry and reverse transcriptase-polymerase chain reaction (RT-PCR)	[152]
*Oxidative stress*	Blood	Plasma oxidized low-density lipoprotein, peroxides, and total equivalent antioxidant capacity (TEAC)	Spectrophotometer	[32]
*Global oxidation*	Blood	plasma fluorescent oxidation products	Spectrophotometry	[40]
*Oxidative stress*	Blood	carboxymethyllysine (CML), advanced glycation end products (AGE)	Spectrophotometry	[40]
*Oxidative stress*	Prostatebiopsy (needle biopsy)	Total thiol groups (TTG) level	Spectrophotometry (2 thionitrobenzoic acid (DTNB))	[13, 153]
*Lymphocyte DNA damage*	Blood	lymphocyte DNA damage	single cell alkaline gel electrophoresis, tail length migration	[15]
*Lipid peroxidation*	Plasma	Thiobarbituric acid reactive substances (TBARS), serum protein carbonylation	Spectrophotometry; Thiobarbituric acid (TBA), concentrations of TBA- MDA adduct	[12, 36, 154]
*Lipid oxidation*	Urine, blood, tissues	F2-isoprostanes	Gas/liquid chromatography-mass spectrometry, mass spectrometry, immunological methods	[40, 61, 155]
*Body composition*	Air	Volume of air the body displaced inside an enclosed chamber (plethysmograph)	Air plethysmography (BOD POD)	[13, 156]

These biomarkers can be assayed in different body specimens such as the whole blood, plasma, serum, urine, and tissue biopsies. Biomarkers discussed in this review are limited to those that are linked to oxidative stress. Recently, F2-isoprostanes, a group of prostaglandin F(2)-like compounds derived from the non-enzymatic oxidation of arachidonic acid have been used as markers of lipid oxidation and the results showed an accurate assessment of oxidative stress both in vitro and in vivo in urine, blood and tissue biopsies [61]. Other biomarkers of oxidative stress are plasma fluorescent oxidation products which detect global oxidation, and carboxy methyl lysine (CML) which detects advanced glycation end products. Higher levels of plasma CML signify increased risk of prostate cancer [40]. Lipid peroxidation contribute to cellular oxidative stress and cell death. This draws attention to the cellular activities that involve the thiol groups. The level of total thiol groups (TTG) has been associated with aging and progression of PCa. A comparison of the levels of TTG between BPH and PCa patients has shown that aging influences a progressive reduction of TTG in BPH patients, while in PCa patients the glutathione concentrations are significantly lower [13]. The determination of the level of 8-isoprostanes (8-EPI) in urine using competitive enzyme-linked immunoassay gives an idea of the oxidative stress status and the stage of the hyperplasia (NIH categories IIIa, IIIb, and IV) [62]. The new phase of diagnosis is moving towards the application of genetics and molecular techniques such as genetic linkage studies which is used to study the expression patterns of ncRNA transcription [63].

### Prostate cancer and benign prostatic hyperplasia (BPH)

These two forms of prostatic hyperplasia are leading cause of urologic problems in older men. Although they can co-exist in an individual, there is no evidence that BPH is a precursor nor risk factor of PCa. There is no evidence that BPH can transform into PCa. However, both conditions share certain attributes in common. They are hormone and age dependent. Risk increases with age, and they are associated with certain forms of hyperplasia [64, 65]. They present similar symptoms and for accurate diagnosis PCa has to be differentiated from BPH. Both PCa and BPH can cause lower urinary tract symptoms (LUTS). Additional tests and examinations are performed to differentiate LUTS caused by PCA and LUTS caused by BPH. For instance, PSA is performed in asymptomatic patients, while prostate biopsy is done on LUTS patients. Procedures such as bone pain are performed on patients with symptoms of metastasis. Evaluating the size of the prostate through digital rectal examination (DRE) is essential in the management of BPH. A combination of DRE and PSA testing can be used to differentiate clinically between PCa and BPH. The presence of a nodular abnormality puts the probability of diagnosing PCa on biopsy at 50 %. Blood level of PSA is no longer used alone for PCa diagnosis. It is now recommended to confirm increases in serum PSA levels with histology tests. Clinically, PCa could be ruled out if PSA and DRE density are normal [64]. Oxidative stress influences the pathophysiology of both PCa and BPH. For instance, antioxidant defense system is decreased in the elderly patients with PCa and BPH [66]. Also, systemic metabolic stress occurs in glucose and fatty acid metabolism in BPH and PCa [67]. However, their response to therapeutics agents differs. And some drugs used in the treatment of BPH have been suspected to worsen the prognosis of PCa. An example is 5α-Reductase inhibitors (5-ARIs) which is the common drug of choice for BPH. It is suspected that 5-ARIs can trigger or increase the risk of developing high-grade prostate cancer. Studies could not associate 5-ARIs use with an increased risk of PCa [37, 68]. Rather in their review, Hamilton and Parsons suggested that 5-ARIs has protective potential and could reduce the risk of prostate cancer in some men [69].

### Prostate cancer, oxidative stress, and age

There is a strong connection between age, OS, and PCA. PCA is predominant among older men and this makes age and family history the strongest predictors of prostate cancer risk. PCa is mostly seen in older men and Black/African Americans are disproportionately affected [20]. The risk of developing PCa increases exponentially with age [15]. For instance, only 1 in 10,000 under age 40 will be diagnosed, the rate goes up to 1 in 38 for ages 40 to 59, and 1 in 14 for ages 60 to 69. Most PCa are diagnosed in men 50 years of age and older [1]. But the age of diagnosis is earlier among Black/African Americans and they are about four times more likely to die from PCa than White men [20, 70]. On the other hand, according to the free radical theory of aging, oxidative stress is blamed for aging because of the negative impact the excessive free radicals or reactive oxygen species (ROS) have on the cells [71]. Also, antioxidant defense system is decreased in the elderly patients with PCa [66]. ROS and age are predominant factors in PCa and other aging-related diseases, such as, diabetes, atherosclerosis and degenerative diseases like Parkinson’s and Alzheimer’s. This is because older cells seem to be more susceptible to intracellular conditions that produce excess ROS which trigger and accelerate tumorigenesis [72]. It has been reported that age increases the prooxidant-antioxidant balance toward a more oxidative state in many tissues [15].

### Prostate cancer and non-coding RNA (ncRNAs)

The interest in getting a better understanding of the molecular mechanisms involved in PCa pathogenesis and prognosis has led to exploring the role of non-coding RNA (ncRNAs). Only about 2 % of human genome are protein-coding sequences while the remaining 98 % are noncoding sequences that can be transcribed in ncRNAs. ncRNAs depending on the size are further grouped into long noncoding RNAs (lncRNAs) and small ncRNAs. Small ncRNAs include microRNAs (miRNAs), piwi-interacting RNAs (piRNAs), ribosomal RNAs (rRNAs), small Cajal body-specific RNAs (scaRNAs), small interfering RNAs (siRNAs), small nuclear RNAs (snRNAs), small nucleolar RNAs (snRNAs) and transfer RNAs (tRNAs). However, miRNAs can also be derived from lncRNAs and snoRNAs [63]. Previously, no functions were apportioned to ncRNAs since they are non-protein-coding RNA species of the transcriptome. Recent studies have revealed that they play special roles in the tumorigenic processes by acting sometimes as oncogenic and tumor suppressor genes. Some perform similar functions as the house keeping genes being involved in mRNA processing and protein transcription [63]. Some ncRNAs perform regulatory functions such as pre- and posttranscriptional gene regulation and chromatin assembly. They are believed to be involved in carcinogenic process by promoting tumor cell proliferation, inducing replicative immortality, encouraging evasion of growth suppressors, stimulating angiogenesis and promoting invasion and metastasis [73]. Aside their role in cancer initiation and progression, non-coding RNA can also be exploited as possible biomarkers of PCa diagnosis, and drug development [74]. There is evidence that ncRNAs are aberrantly expressed in prostate cancer and carcinogenesis and tumor progression may result from series of multicellular activities due to dysregulation of ncRNA controlled pathways [74].

In PCa some lncRNAs are dysregulated and are emerging as major biomarkers of cancer development and therapeutic targets. For example, PCA3, PCATs, SChLAP1, SPRY4-IT1 and TRPM2-AS are lncRNAs upregulated in PCa [75]. Other studies have demonstrated the role of lncRNAs in regulatory and cellular processes including chromatin modification, alternative splicing, post-transcriptional processing and cell signaling transduction [76]. Dysregulation of their function may have deleterious effects by inducing chromosomal translocation, deletion, and nucleotide expansions. Recent studies demonstrate that multiple prostate cancer risk loci are associated with lncRNAs and that ectopic expression of these transcripts triggers a cascade of cellular events driving tumor initiation and progression [24]. The discovery of the lncRNA prostate cancer antigen 3 (PCA3, or DD3), which is specifically overexpressed in malignant prostate tissue supports the claim that lncRNAs may have cancer-specific expression. These additional lnRNAs AK024556, XLOC_007697, LOC100287482, XLOC_005327, XLOC_008559, and XLOC_009911 which are differentially expressed in prostatic adenocarcinoma tissue samples could be possible biomarker targets of PCa [77].

### Prostate cancer and microRNA

MicroRNAs (miRNA) are small, non-coding, single-stranded, short nucleotide sequences (between 19 and 25 nucleotides long) that be derived from lncRNAs and snoRNAs [63]. miRNAs function as regulators of gene expression and influence various physiological and pathophysiological processes [78] by binding to targeted messenger RNA (mRNA) sequences post-transcriptionally through complementary binding and modulate gene expression. They can control gene expression by silencing or degrading targeted mRNA [79]. Deregulation of miRNA in PCa may contribute to cancer initiation and metastatic progression [69]. miRNA can be good biomarker in diagnosing and predicting prognostic outcomes of PCa as miR-148a is differentially expressed in PCa [69]. Identification of dysregulated microRNAs (miRNAs) in prostate cancer is critical not only for diagnosis, but also differentiation between the aggressive and indolent forms of the disease. Some miRNAs modulate the activities of mesenchymal stem cells (MSCs) especially adipose-derived stromal cell (ASC) that has therapeutic effects on PCa. A novel miR-145 is involved in PCa cell apoptosis cell induction by mediating the inhibitory effect of ASC on PCa [80]. Understanding the expression patterns of circulating miRNAs and correlating them with disease status could offer an alternative minimally invasive approach to monitor the prognosis of PCa progression [81].

Deregulation of miRNA expression could also have positive outcomes; for example inhibition of miR-9 can reduce tumor growth and metastases by slowing down the migratory and invasive potential of the M12 cell line. miR-9 modulates the expression of e-cadherin and suppressor of cytokine signaling 5 (SOCS5) which cancer linked proteins [82]. Another miRNA, miR-199a-3p targets stemness-related and mitogenic signaling pathways which suppresses the expansion and tumorigenic capabilities of prostate cancer stem cells and aberrant loss of a miRNA-mediated mechanism can lead to the expansion and tumorigenic activity of prostate cancer stem cells (CSCs) [83]. The use of miRNA to silence genes involved in PCa tumorigenesis such as STAMP2 required for PCa progression could be a viable therapeutic mechanism for PCa [40].

Circulating miRNAs could be non-invasive markers of disease and can be used as biomarkers in PCa diagnosis. For example, determining the levels of 15 miRNAs and miR-141 could be used to differentiate metastatic PCa patients from healthy subjects [84] or between BPH and PCa [85]. But some of the miRNAs are not specific for a particular cancer type and thus, serum miRNAs may distinguish between different cancer types [86]. But miR-21 appears to be elevated in CRPC patients who are resistant to docetaxel than in BPH. While miR-150 enhances targeted endothelial cell migration [87]. miR-26a, miR-195, and let-7i levels were elevated in PCa compared to BPH samples [85]. Other studies have reported additional miRNAs that can be used in PCa diagnosis. Moltzahn identified 7 miRNAs differentially expressed between PCa patients and healthy subjects [88]. For instance, miR-141, and miR-375 expression are elevated in PCa and their presence in the blood stream may serve as evidence of advanced cancer disease [89]. Further, miR-141 levels have been associated with clinical progression and it is positively correlated with PSA [90]. Both miR-21, miR-221 [91] and miR-141 levels were elevated in PCa patients compared to healthy controls but the levels were higher in metastatic PCa than in localized tumors [92]. PCa progression could be monitored by measuring the elevation levels of hsa-miR-141, hsa-miR-298 and hsa-miR-375 [93]. Monitoring urinary levels of miR-107 and miR-574-3p could also be relevant in PCa diagnosis [94]. The diagnostic reliability of miRNA in PCa could be further improved by identifying a panel of miRNAs instead of single miRNA. Chen and co-workers identified a panel of 5miRNAin this regard; let-7c, let-7e, miR-30c, miR-622, and miR-1285. According to them this panel could differentiate between PCa patients and healthy people and between PCa and BPH [95]. miR-20a, miR-21, miR-145, and miR-221 can be associated with tumor risk scores [96] while SNORD43 may be a suitable reference gene for the analysis of circulating miRNA in patients with urological malignancies [97].

### Prostate cancer and inflammatory oxidative stress

Prostatic inflammation is suggested to be involved in the pathogenesis and progression PCa [98]. Inflammation is thought to incite carcinogenesis by causing cell and genome damage, promoting cellular turnover [99]. Prostatic inflammation could also be caused by bacterial infections, urine reflux, dietary factors, hormones, and autoimmune response [3]. Irregularities in the functioning of genes involved in oxidative stress have been linked to inflammatory response in PCa. For instance the loss or aberration in the expression of glutathione S-transferase P1 (GSTP1) may contribute to the transition of proliferative inflammatory atrophy (PIA) into high-grade intraepithelial neoplasia (HGIPN) and PCa in patients with genetic predisposition [3].

Inflammation is known to trigger cytokines production which contributes to tumorigenesis in different tissues. For example, prostaglandin endoperoxide synthase 2, also referred to as cyclooxygenase 2 (COX-2), is an enzyme involved in the conversion of arachidonic acid to prostaglandins and other eicosanoids. The overexpression of COX-2 has been reported to cause phenotypic changes in intestinal epithelial cells that could enhance their tumorigenic potential [100]. COX-2 as a proinflammatory cytokine may also create an environment that favors local growth factor production and angiogenesis in the prostatic tissue [78]. Oxidative stress ensues when the proinflammatory microenvironment creates a local hypoxia induced by increased oxygen demands by proliferating cells leading to tissue injury in infiltrating area [3] (Fig. 4). The literature contains numerous studies associating PCa and inflammation but none has categorically established a causal relation. Intake of anti-inflammatory drugs and antioxidants leads to a decrease in PCa risk [18]. Further understanding of the role of inflammation in oxidative stress induction and PCa promotion and progression is necessary as it may revolutionize the way PCa is treated [16].

### Oxidative stress and DNA damage in prostatic hyperplasia

A genetic predisposition or acquired genetic and epigenetic changes with effect other factors, such as advanced age, race and environmental factors contribute to PCa development [53]. OS triggers metabolic reprogramming responsible for malignant transformation and tumor development, including invasion and metastasis [101]. The activities of antioxidant enzymes and the levels of antioxidant, reduced glutathione have been found to be significantly decreased in prostatic hyperplasia. Significantly increased levels of oxidative stress and DNA damage suggest that oxidative damage plays an important role in prostate tumorigenesis and timely management of oxidative stress can be of importance in preventing the occurrence of prostatic hyperplasia [11, 16]. Other studies have revealed that oxidative stress mediated pathways are involved in several male urologic disorders including the different forms of prostatitis (NIH categories IIIa, IIIb, and IV). The new insight into the role of oxidative stress in the pathogenesis of PCa has led to the exploitation of this mechanism as a potential strategic target for PCa treatment [30, 62].

### Molecular biology of oxidative stress and prostate cancer

More knowledge about the etiology and progression of PCa has been provided by the advancement in molecular biology and development of new techniques such as microarray [102] and RNAseq gene expression, genome-wide linkage analysis, and loss of heterozygosity (LOH) [103]. Recently developed methods for profiling genome-wide occupancy of lncRNAs have allowed high-throughput identification of RNA–DNA and RNA–protein interactions. Two methods called Chromatin Isolation by RNA Purification (ChIRP) [104], Native RNA immunoprecipitations sequence(RIP-seq) [105] and CHART, that use complementary oligonucleotides to pull down lncRNAs associated with chromatin, have been developed to determine the chromatin binding sites for lncRNAs.68 Alternatively, RNA immunoprecipitation sequencing (RIP-Seq) and photoactivatable ribonucleoside-enhanced crosslinking and immunoprecipitation (PAR-CLIP) represent complementary approaches to the study of RNA–protein interactions.69,70 These new techniques represent promising tools to explore the mechanisms that govern lncRNA-chromatin interactions, as shown by the informative analyses performed to date on select lncRNAs. Other techniques that have been applied in PCa studies include methods which identify microsatellites, single nucleotide polymorphism (SNP) and haplotype mapping to monitor the distribution of clusters of SNPs that segregate together in linkage disequilibrium [106]. These techniques have revealed the potential role of DNA repair genes, tumor suppressor genes, oncogenes, and protein expression in the prognosis, diagnosis, and possible therapeutic strategies for PCa. Also, linkage analyses in genome wide studies have given clues as to why the disease runs in a family and why a particular race may be at higher risk of developing and dying from the disease [103, 107–111].

Only about 9 % of PCa cases are linked with heredity and studies are carried using multipoint linkage analyses with microsatellite markers. These studies have identified PCa susceptibility loci on chromosome 1, including hereditary PCa families (HPC), HPC1 (1q24–q25), PCAP (1q42–q43), HPCX (Xq27–q28), CAPB (1p36), HPC20 (20q13), HPC2/ELAC2 (17p11) and 16q23 [107, 108, 111]. And based on reports 5q31–q33, 7q32 and 19q12 were described as prostate cancer aggressiveness loci [103]. Although inconsistent results were obtained from repeated linkage studies of these regions, HPC1 is thought be common among people with early onset disease. Nevertheless, hereditary of PCa is heterogeneous and there are multiple loci on chromosome 1 for this disease [103, 107, 108].

Genetics is a major determinant of susceptibility to PCa on a wide population [112]. Aberrations in gene expression and gene mutations have been seen in prostate cancer including PTEN, KAI1, SRD5A2, and IL6 and they are associated with PCa progression [113]. Genomewide studies have revealed multiple loci that can bind to PCa candidate susceptibility genes such as MSMB, LMTK2 and KLK3, CPNE3, IL16 and CDH13. For example MSMB which encodes beta-microseminoprotein, a primary constituent of semen and a potential prostate cancer biomarker, and CTBP2, a gene with antiapoptotic activity are located on chromosome 10 [109, 110]. Genes associated with the redox homeostasis of the cell are being reported to undergo transformation that influences susceptibility to PCa. Of particular interest is the six transmembrane protein of prostate 2 (STAMP2) which is an androgen-regulated gene whose mRNA expression is increased in PCa. The STAMP2 protein expression is increased in human PCa. It is suggested that STAMP2 also significantly increases reactive oxygen species (ROS) in PCa cells because its iron reductase activity has the ability to deplete NADPH levels [40].

A search for prostate cancer/molecular biology on Cancer Index Web Resource and NCBI [50, 113] was used to construct Table 2 which shows a list of genes that are associated with PCa based on scientific publications. The table is not exhaustive and most of genes are known to regulate critical mechanisms in other types of cancer. The list includes the gene symbols, gene names, and their location on the chromosome and the number of scientific publications on each of the genes (Table 2). Genes that regulate critical mechanisms in other cancers and which are expressed in prostate cancer include Kallikrein-related peptidase 3 (KLK3), also referred to as prostate surface antigen (PSA) [50, 113]. PSA is elevated in PCa and serum measurement of PSA is a common diagnostic test for diagnosis and monitoring of PCa in hospital settings [114]. CD82 molecule, a metastasis suppressor gene product known to be downregulated in tumor progression of human cancers and can be activated by p53. It is co-expressed with p53 and aberration in CD82 expression is linked with poor survival for PCa patients [115]. Another gene in Table 2 is microseminoprotein, beta (MSMB) which encodes a protein that belongs to the immunoglobulin binding factor family. The protein is synthesized by the epithelial cells of the prostate gland and secreted into the seminal plasma. This protein has inhibin-like activity and its expression decreases in PCa [116, 117]. A tumor suppressor gene, Ras association (RalGDS/AF-6) domain family member 1 (RASSF1) has been seen in other cancers and is believed to play a role in PCa pathogenesis [118]. Clusterin on the other hand encodes a protein that regulates several basic biological events including cell death, tumor progression, and neurodegenerative disorders [119]. NK3 homeobox 1 (NKX3-1) encodes a homeobox-containing transcription factor which serves as a negative regulator of epithelial cell growth in prostate tissue. Aberrant expression of this gene is associated with prostate tumor progression [111].

**Table 2:** List of Genes Associated with Prostate Cancer and Oxidative Stress. [50, 113]

Gene	Gene name	Location	Papers
KLK3	Kallikrein-related peptidase 3	19q13.41	3000
AR	Androgen receptor	Xq12	1221
MKI67	Marker of proliferation Ki-67	10q26.2	424
PTEN	Phosphatase and tensin homolog	10q23.3	376
TP53	Tumor protein p53	17p13.1	343
TMPRSS2	Transmembrane protease, serine 2	21q22.3	336
CTNNB1	Catenin (cadherin-associated protein), beta 1	3p21	312
BRCA1	Breast cancer 1, early onset	17q21	178
BRCA2	Breast cancer 2, early onset	13q12.3	156
PROC	Protein C (inactivator of coagulation factors Va and VIIIa)	2q13-q14	135
CDKN1A	Cyclin-dependent kinase inhibitor 1A (p21, Cip1)	6p21.2	125
NKX3-1	NK3 homeobox 1	8p21.2	120
SRD5A2	Steroid-5-alpha-reductase,	2p23	120
SRC	SRC proto-oncogene, non-receptor tyrosine kinase	20q12-q13	97
KITLG	KIT ligand	12q22	93
CDKN1B	Cyclin-dependent kinase inhibitor 1B (p27, Kip1)	12p13.1-p12	92
CD44	CD44 molecule (Indian blood group)	11p13	87
TGFB1	Transforming growth factor, beta 1	19q13.1	83
HIF1A	Hypoxia inducible factor 1, alpha subunit (basic helix-loop-helix transcription factor)	14q23.2	82
CYP17A1	Cytochrome P450, family 17, subfamily A, polypeptide 1	10q24.3	82
PTGS2	Prostaglandin-endoperoxide synthase 2 (prostaglandin G/H synthase and cyclooxygenase)	1q25.2-q25.3	81
PPARG	Peroxisome proliferator-activated receptor gamma	3p25	81
PCA3	Prostate cancer associated 3 (non-protein coding)	9q21.2	78
IGFBP3	Insulin-like growth factor binding protein 3	7p12.3	76
EZH2	Enhancer of zeste 2 polycomb repressive complex 2 subunit	7q35-q36	75
ETV1	ETS (E twenty-six) variant 1	7p21.3	73
GSTM1	Glutathione S-transferase mu 1	1p13.3	72
JUN	Jun proto-oncogene	1p32-p31	65
CAMP	Cathelicidin antimicrobial peptide	3p21.3	61
ELAC2	ElaC ribonuclease Z 2	17p11.2	56
SERPINB5	Serpin peptidase inhibitor, clade B (ovalbumin), member 5	18q21.33	54
CD82	CD82 molecule	11p11.2	50
AMACR	Alpha-methylacyl-CoA racemase	5p13	50
IGF1R	Insulin-like growth factor 1 receptor	15q26.3	49
IL10	Interleukin 10	1q31-q32	47
E2F1	E2F transcription factor 1	20q11.2	46
MSMB	Microseminoprotein, beta- (10q11.2)	10q11.2	45
TRPM2	Transient receptor potential cation channel, subfamily M, member 2	21q22.3	44
CYP3A4	Cytochrome P450, family 3, subfamily A, polypeptide 4	7q21.1	43
CLU	Clusterin	8p21-p12	43
PSCA	Prostate stem cell antigen	8q24.2	42
FOS	FBJ murine osteosarcoma viral oncogene homolog	14q24.3	42
CASP9	Cysteine-aspartic acid protease (caspase) 9, apoptosis-related cysteine peptidase	1p36.21	40
VEGFA	Vascular endothelial growth factor A	6p12	40
FOXA1	Forkhead box A1	14q21.1	40
MET	MET proto-oncogene, receptor tyrosine kinase	7q31	40
CYP3A5	Cytochrome P450, family 3, subfamily A, polypeptide 5	7q21.1	39
RASSF1	Ras association (RalGDS/AF-6) domain family member 1	3p21.3	39
PDLIM4	PDZ and LIM domain 4	5q31.1	38
MSR1	Macrophage scavenger receptor 1	8p22	38

Prostate stem cell antigen (PSCA) encodes a glycosylphosphatidylinositol-anchored cell membrane glycoprotein and it is secreted in the prostate as well as in other organs such as the colon and pancreas. PSCA is up-regulated in a large proportion in PCa and is also detected in cancers of the bladder and pancreas [120]. Pten is also associated with different types of malignancies and it is known to be involved in PCa development where it coordinates the differentiation and proliferation of cell types [121]. ELAC ribonuclease 2 (ELAC2) functions as a transcription factor. It interacts with activated Smad family member 2 (Smad2) and its nuclear partner forkhead box H1 (FAST-1). Mutations in this gene may lead to an increased risk of PCa [122, 123]. PCa-associated 3 (PCA3) produces a spliced, long non-coding RNA that is highly overexpressed in most types of prostate cancer cells and is used as a specific biomarker for this type of cancer [83]. Glutathione S-transferase mu 1 (GSTM1) is involved in detoxifying electrophilic compounds and neutralizing the effect of anti-oxidants in the cellular system, glutathione conjugates with the products of oxidative stress [124, 125]. Prostaglandin-endoperoxide synthase 2 (PTGS), also known as cyclooxygenase, is the key enzyme in prostaglandin biosynthesis, and acts both as a dioxygenase and as a peroxidase. This gene encodes inducible isozyme which induces prostanoid biosynthesis. Prostanoid is thought to be involved in inflammation and mitogenesis [124]. Hypoxia inducible factor 1, alpha subunit (HIF1A) encodes the alpha subunit of transcription factor hypoxia-inducible factor-1 (HIF-1), which is a heterodimer consisting of an alpha and a beta subunit. HIF-1 is involved in the regulation of cellular and systemic homeostatic response to hypoxia. It activates transcription of genes involved in energy metabolism, angiogenesis, apoptosis, and other genes that secret protein which promote metabolic adaptation to hypoxic environment as well as increase oxygen delivery. It may also be involved in the activation of signaling pathways. Redox-sensitive transcription factors like HIF-1α has been shown to play a major role in progression and metastasis of the cancer cells [126]. Steroid-5-alpha-reductase (SRD5A2) gene encodes a microsomal protein expressed at high levels in androgen-sensitive tissues such as the prostate and male pseudohermaphroditism, specifically pseudovaginal perineoscrotal hypospadias may occur if the gene is suppressed or deficient [127].

Cellular dysfunction resulting from polymorphisms and post translation modifications such as methylation in genes contributes to PCa risk. For instance, changes in the glutathione S-transferase (GST) genes have been implicated as risk factors for prostate cancer [128]. Loss of GSTP1 expression via promoter hypermethylation is the most common epigenetic alteration observed in human PCa. Dysfunction of GSTP1, a member of the GST gene family, can trigger an increased production of reactive oxygen species (ROS) and DNA damage in cells. Thus, monitoring GSTP1 expression in human prostate cells may be an important target for primary prevention of PCa knowing that in its absence cells are more prone to oxidative stress induced DNA damage and cell death [129]. The GST superfamily consists of four gene classes (A, M, T, and P) encoding for enzymes which catalyze the conjugation of electrophilic compounds to glutathione [130]. These enzymes are also believed to play a crucial role in the protection of DNA from oxidative damage [131]. The pi-class glutathione S-transferase (GSTP1) actively protect cells from carcinogens and electrophilic compounds. Previous studies have shown that the CpG-rich promoter region of the pi-class gene GSTP1 is methylated at single restriction sites in the most prostate cancers [132, 133]. It has been observed that in normal prostate tissue the entire CpG island is unmethylated but outside the island in the body of the gene is highly methylated. The DNA methylation of the CpG island in both PCa cell lines and cancer tissues occurs when GSTP1 expression is repressed [128]. Tumor suppressor genes and other CpG island-containing genes such as calcitonin, p15, p16, Rb, VHL, e-cadherin, ER, and HIC1 have been found in the hypermethylated region [97].

**Fig. 5:**
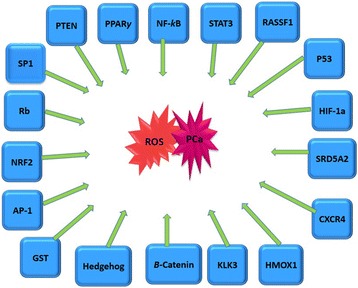
Oxidative Linked Genes Involved in Prostate Cancer: These genes have been linked to oxidative stress and their expression was aberrant in prostate cancer [26, 151]

There are other polymorphs of GST such as the mu (GSTM1) and theta (GSTT1) which regulate the conjugation of carcinogenic compounds to excretable hydrophilic metabolites making the genes more susceptible to various carcinogens. Changes in their structure such as double deletion (GSTM1-/GSTT1-) is associated with higher oxidative stress which might exacerbate the pathogenesis of BPH and PCa [36]. In normal prostate, the transduction pathway from NIK to NF-kB seems to be inactive. However, in BPH, it has been reported that TNF-α/AP-1 transduction pathway is activated followed by a stimulation of the apoptotic pathway to inhibit uncontrolled cell proliferation [134]. Another study has also demonstrated a novel link between OS and loss of imprinting, showing that OS as measured by the increase in NF-kB activity, induces loss of imprinting of insulin-like growth factor 2 in both cancerous and noncancerous human prostate cells (Fig. 5). This loss during aging contributes to tumorigenesis and NF-kB modulation is important as it may prevent age-related alteration in the epigenome [24].

**Fig. 6:**
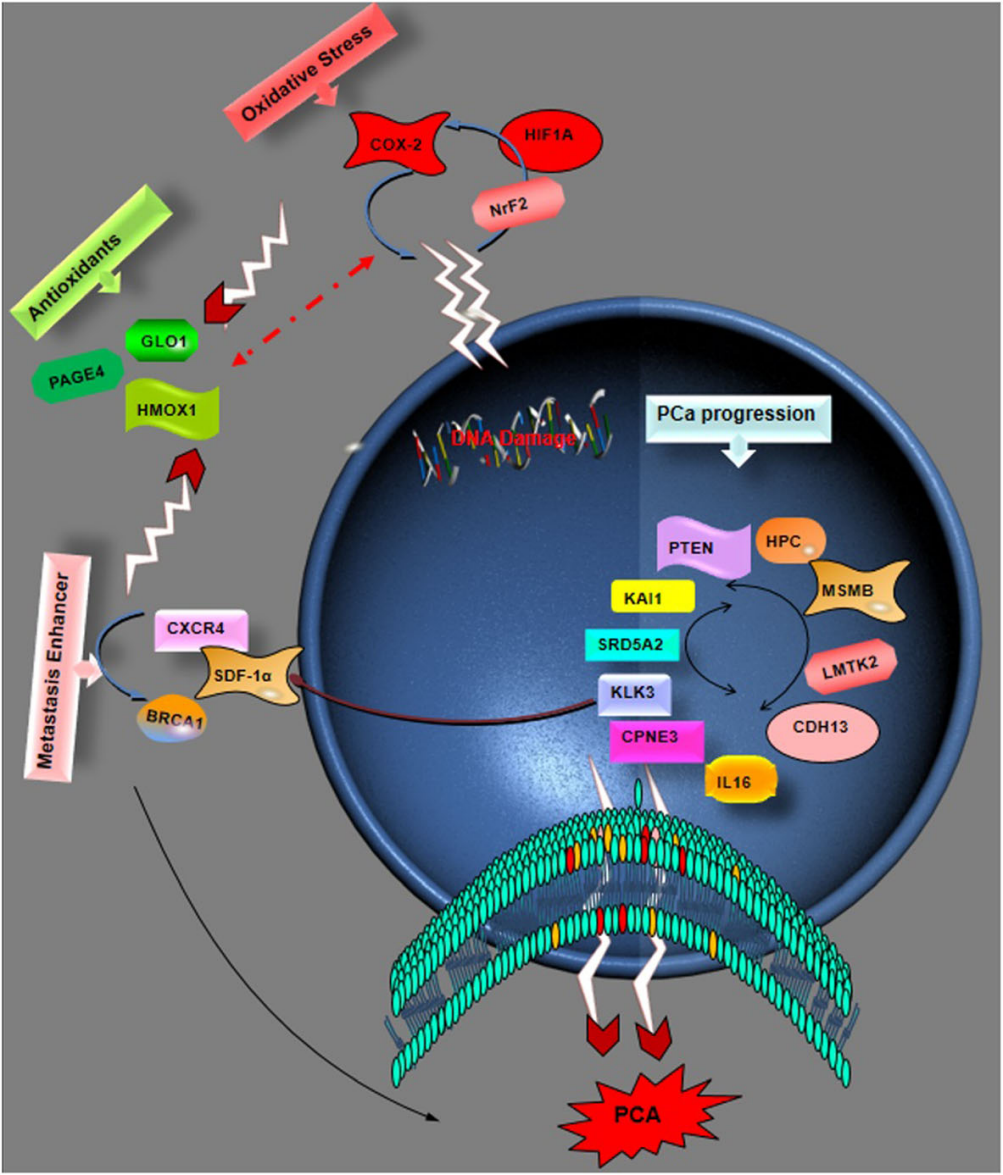
Gene Involved in the signaling pathways which contribute to the development and progression (including invasion, metastasis, and relapse) of PCa: The genes that maintain hemostasis receive attack from pro-oxidative stress genes and genes that are involved in metastasis. DNA damage leads to suppression of antioxidant pro-gene and this gives way to the over-expression of cancer promoting genes

PCa tumorigenesis involves a combo of genes such as the antioxidant enzyme heme oxygenase 1 (HMOX1/HO-1) which is responsible for the maintenance of the cellular homeostasis (See Fig. 6). HMOX1/HO-1 plays a critical role in oxidative stress mechanism and in the regulation of PCa development and progression. The transcription factor Nrf2 and BRCA1 protein synergistically activate HO-1 promoter activity forming BRCA1-Nrf2/HO-1 which function in the maintenance of the cellular homeostasis in PCa. They exert both oxidative and genotoxic stress on HO-1 transcriptional activity [135]. ROS increase the expression and activity of the chemokine receptor, cysteine (C)-X-C Receptor 4 (CXCR4), which enhances metastatic functions in prostate cancer cells. Also, CXCR4 and its ligand, SDF-1α, promote ROS accumulation contributed by the NADPH oxidase (NOX) family of enzymes. NOX2 expression is associated with PCa. CXCR4/SDF-1α-mediated ROS production through NOX2 enzymes may be an emerging concept by which chemokine signaling progresses tumorigenesis [136]. Glyoxalase 1 (GLO1) is a glutathione-dependent enzyme that acts as a scavenging enzyme. It participates in ROS mechanism and is involved in the occurrence and progression of human malignancies. Polymorphism in Glyoxalase I A111E may influence its enzymatic activity. GLO1 could be important in PCa progression and may be a good marker for risk assessment and prognosis in PCa patients [137]. Another gene, prostate-associated gene 4 (PAGE4) encodes a protein which protects cells against stress by elevating p21 and suppressing ROS production. PAGE4 is a cancer/testis antigen (CTA) that is up-regulated in PCa and seen in symptomatic patients. However, PAGE4 appears to protect cells from transforming to PCa by its stress-protective and anti-apoptotic activities [138]. The role of OS and diet in PCa mechanism was observed with the prostate-specific ablation of PPAR*γ* in mice. This action resulted in tumorigenesis and active autophagy. Placing the mice on high-fat diet (HFD) caused downregulation of PPAR*γ*-regulated genes and decreased prostate differentiation. This suggests that systemic metabolic stress occurs in glucose and fatty acid metabolism in benign and PCa [67].

Pyruvate kinase M2 (PKM2) is essential for aerobic glycolysis, the dominant metabolic pathway utilized by cancer cells. To determine the association of PKM2 with prostate cancer (PC). PKM2 was found to be upregulated and undergoes post translational modification in PCa [139]. Pten as shown in Table 2 is involved in PCa cellular programing and signaling and G6PD metabolism. AR signaling can promote prostate cancer through the upregulation of G6PD and thus providing sugars via the pentose phosphate pathway. This suggests there are other metabolic pathways apart from the glycolysis which prostate cancer growth can be promoted and sustained [140].

Dietary factors are considered responsible for the geographical differences in prostate cancer incidence and mortality. Since about 50 % of all men worldwide, from both East and West, show evidence of microscopic cancer by 50 years of age, growth restraint would appear to be the pragmatic option to the possibility of preventing initiation [141].

### Preventive effects of antioxidants against prostate cancer

Published research has shown that the antioxidant defense system is decreased in the elderly patients with PCa and BPH [66]. The 2-hydroxy-4-methoxy benzoic acid (HMBA), is an antioxidant that has been shown to have protective potential against testosterone induced BPH in rats [29]. Chemopreventive effects of the essential trace element selenium against prostate cancer have been shown in preclinical models and human observational studies, but results from clinical trials have been disappointing. It appears that there is a threshold selenium (Se) status below which improvement will decrease PCa risk, but above which supplemental Se may be deleterious. Different forms of selenium have different effects, and genetic and other factors modify selenium's chemopreventive potential [23]. However, there has been reduced interest in pursuing chemoprevention strategies targeting oxidative stress because of the failure of the Selenium and Vitamin E Cancer Prevention Trial (SELECT) [72]. Another promising therapeutic candidate is comprised of plant-derived dietary polyphenolic compounds, such as flavonoids that have cancer cell-specific pro-apoptotic activity and chemopreventive potential. Flavonoid glycosides are also found to be DNA hypomethylating agents with an ability to modulate cancer cell epigenome leading to changes in the gene expression patterns. For example, diosmin which is a dietary flavonoid glycoside was found to be active against DU145 cells by promoting genotoxic events that led to apoptotic cell death [142].

### Oxidative stress, antioxidants and prostate cancer treatment

The classical treatment involves bilateral orchiectomy, or administration of diethylstilbestrol (DES). Other treatment strategies are based on endocrine treatment. The principle for endocrine treatment of prostate cancer is to deprive the cancer cells of androgens. Androgen deprivation is an effective treatment for patients with advanced prostate cancer but it is not curative and creates unwanted side effects [143]. For metastatic PCA, castration is still the best choice of treatment as orchiectomy, oestrogen agonists and GnRH agonists produce equivalent clinical responses [144]. It is interesting to note that maximum androgen blockade (MAB) is not strikingly more effective than single agent GnRH agonist or orchiectomy. However, for locally advanced PCA, nonsteroidal antiandrogen monotherapy is as effective as castration [144].

Although oxidative stress has been associated with several destructive mechanisms in biological systems, induction of oxidative stress can also provide a means for a potent and safe cancer treatment [145]. Oxidative stress has been shown to promote castration resistance via androgen receptor (AR)-dependent pathway such as AR overexpression, AR cofactor, and AR post-translational modification as well as AR-independent pathway, leading to the emergence of castration-resistant PCa (CRPC). Thus antioxidants therapy using natural and chemical ROS scavengers and inhibitors of ROS production seems to be a promising therapy for CRPC as well as preventing castration resistance [146]. Androgen deprivation therapy (ADT) has been reported to lower basal ROS level in prostate cancer (PCa) and to sensitize PCa to radiation. An in vivo experiment with transgenic adenocarcinoma of the mouse prostate (TRAMP) androgen deprivation resulted in an increase in basal ROS level in PCa cells with AR expression. also, the genetic Nrf2 upregulation lowered basal ROS similar to ADT [147].

The major androgen within the prostate is dihydrotestosterone (DHT). DHT and 5α-reductase are highly associated with prostate cancer. It has been hypothesized that inhibition of 5α-reductase activity might reduce the risk of prostate cancer development, slow tumor progression and even treat the existing disease. The therapeutic mechanism of some of the most recommended drugs for prostatic hyperplasia treatment, 5-ARIs, is based on their reductive effect on testosterone, progesterone, androstenedione, cortisol, aldosterone, and deoxycorticosterone [148]. For example, dutasteride (Avodart) suppresses all three 5α-reductase isoenzymes and reduces dihydrotestosterone in men with benign prostatic hyperplasia [149]. Exposure of human PCa cells to KML001 (NaAsO2, sodium metaarsenite, Kominox), an orally bioavailable arsenic compound, induces both apoptotic and autophagic cell death via oxidative stress pathway. Also, KML001 has an antiproliferative effect on DU145 cells in xenograft mice [37]. It has been reported that the therapeutic silencing of STAMP2 by administration of nanoliposomal siRNA profoundly inhibits tumor growth in two established preclinical PCa models in mice. These findings suggest that STAMP2 is required for PCa progression and thus may serve as a novel therapeutic target [40]. There is evidence that αATA (8,24) (3α-acetyloxy-tir-8,24-dien-21-oic acid) inhibits Akt/mammalian target of rapamycin (mTOR) signaling. Compared with related tirucallic acids, αATA (8,24) is the most potent inhibitor of the proliferation of androgen-insensitive PCa cells in vitro and in vivo. In PCa xenografted onto chick chorioallantoic membranes αATA (8,24) induced loss of cell membrane asymmetry, caspase-3 activation, and DNA fragmentation in vitro and in vivo. The ability of αATA (8,24) to inhibit Akt/mTOR signaling and to simultaneously induce oxidative stress could be exploited for the development of novel antitumor therapeutics with a lower profile of toxic side effects [70].

## Conclusions

This review has demonstrated that there are several studies which support the fact that oxidative stress plays a critical role in PCa. This oxidative damage is mediated by an overproduction of oxidant molecules and concomitant deficiency in the antioxidant system response which causes an imbalance in the cell redox homeostasis. PCa is associated with both age, and oxidative stress which also contributes to the aging process. Oxidative stress affects all aspects of cellular functions and processes including DNA replication and cell division, and inflammatory responses. Both endogenous and exogenous antioxidants contribute to the reduction of PCa by neutralizing the damaging effect of oxidative stress. However, the oxidative mechanism can also be exploited for treatment of prostate related diseases including PCa.

## Abbreviations

3αATA (8,24): 3α-acetyloxy-tir-8,24-dien-21-oic acid
5-ARIs: 5α-Reductase inhibitors
5-ARIs: 5α-reductase inhibitors
8-EPI: 8-isoprostanes
8-OHdG: 8-hydroxy-2'-deoxyguanosine
ADT: Androgen deprivation therapy
Akt: Alpha serine/threonine-protein kinase
AP1: Activator protein 1
AR: Androgen receptor
BHP: Benign prostatic hyperplasia
BRACA1: Breast cancer 1 gene
CDH13: Cadherin13
CML: Carboxy methyl lysine
COX2: Cyclooxygenase 2
CPNE2: Calcium-dependent membrane-binding protein
CPPS: Chronic pelvic pain syndrome
CRPC: Castration-resistant prostate cancer
CTA: Cancer/testis antigen
CXCR4: Chemokine receptor type 4
DNA: Deoxyribonucleic acid
DRE: Digital rectal examination
ELAC2: ElaC ribonuclease Z 2
ER: Estrogen receptor
FM: Fat mass
GLO1: Glyoxalase 1
GPx: Glutathione peroxidase
GST: Glutathione S-transferase
HFD: High-fat diet
HGIPN: High-grade intraepithelial neoplasia
HIC1: Hypermethylated in cancer 1 gene
HIF1A: Hypoxia inducible factor 1, alpha subunit
HMBA: 2-hydroxy-4-methoxy benzoic acid
HMOX1: Heme oxygenase 1
HO-1: Heme-oxygenase-1
HPC: Hereditary prostate cancer
IL6: Interleukin 6
iNOS: Inducible nitric oxide synthase
KLK3: Kallikrein-related peptidase 3
LOH: Loss of heterozygosity
LSMK2: Lemur tyrosine kinase 2
LUTS: Lower urinary tract symptoms
MDA: Malondialdehyde
MnSOD: Manganese superoxide dismutase
MSMB: Microseminoprotein, beta
mTOR: Mammalian target of rapamycin
NADPH: Nicotinamide adenine dinucleotide phosphate
NF-kB: Nuclear factor kappa B
NIH: National Institutes of Health
NIK: Nuclear factor kappa B inducing kinase
NOX: NADPH oxidase
Nrf2: Nuclear factor erythroid 2–related factor 2
OS: Oxidative stress
PAGE4: Prostate-associated gene 4
PCa: Prostate cancer
PEN: Phosphatase and tensin homolog
PH: Prostatic hyperplasia
PIA: Proliferative inflammatory atrophy
PIN: Prostate intraepithelial neoplasia
PKM2: Pyruvate kinase M2
PPARγ: Peroxisome proliferator-activated receptor gamma
PSCA: Prostate stem cell antigen
PTGS: Prostaglandin-endoperoxide synthase 2
Rb: Retinoblastoma
RNASeq: Ribonucleic acid sequencing
RNS: Reactive nitrogen species
ROS: Reactive oxygen species
SDS 1: Stromal cell-derived factor 1
SNP: Single nucleotide polymorphism
SOD: Superoxide dismutase
SRD4A2: 3-oxo-5-alpha-steroid 4-dehydrogenase 2
STAMP2: Six transmembrane protein of prostate 2
STAT3: Signal transducer and activator of transcription 3
TBARS: Thiobarbituric acid reactive substances
TNF: Tumor necrotic factor
TRAMP: Transgenic adenocarcinoma of the mouse prostate
Trx1: Thioredoxin 1
TTG: Total thiol groups
VHL: Von Hippel–Lindau syndrome
WC: Waist circumference
